# The time course of erythrocyte membrane fatty acid concentrations during and after treatment of non-human primates with increasing doses of an omega-3 rich phospholipid preparation derived from krill-oil

**DOI:** 10.1186/s12944-017-0414-9

**Published:** 2017-01-21

**Authors:** Petter-Arnt Hals, Xiaoli Wang, Fabiana Piscitelli, Vincenzo Di Marzo, Yong-Fu Xiao

**Affiliations:** 1 Aker Biomarine Antarctic AS, Oksenoyveien 10, N-1366 Lysaker, Norway; 2 Crown Bioscience (Taicang) Inc., Science and Technology Park, 6 Beijing West Road, Taicang, Jiangsu Province People’s Republic of China; 3 Endocannabinoid Research Group, Institute of Biomolecular Chemistry, Consiglio Nazionale delle Ricerche, Pozzuoli, NA Italy

**Keywords:** Phospholipids, Long-chain omega-3 fatty acids, EPA, DHA, Omega-3 index, Endocannabinoids

## Abstract

**Background:**

A commonly used measure to reflect the intake of the long-chain omega-3 fatty acids EPA and DHA is the omega-3 index, defined as the sum of EPA + DHA as % of total fatty acids in erythrocyte membrane. When the omega-3 index changes it follows that the relative fractions of other fatty acids in the membrane are also changed. In the present study, increasing doses of a preparation of omega-3 rich phospholipids extracted from krill oil were administered orally to non-human primates for 12 weeks and the time course of EPA, DHA and 22 other fatty acids in erythrocytes was determined bi-weekly during treatment and for 8 weeks after cessation of treatment. Plasma concentrations of six endocannabinoid-type mediators being downstream metabolites of some fatty acids analyzed in erythrocytes were also determined.

**Methods:**

Six diabetic, dyslipidemic non-human primates were included, three in a vehicle control group and three being treated with the omega-3 rich phospholipid preparation. The vehicle control and test items were given daily by gavage and the test item doses were 50, 150 and 450 mg phospholipids/kg/day. Each dose level was given for four weeks. Blood was sampled at baseline and thereafter bi-weekly. Fatty acids were determined in erythrocytes by methylation followed by gas-chromatography. Endocannabinoids and endocannabinoid-like mediators were analyzed in plasma by liquid chromatography-atmospheric pressure chemical ionization-mass spectrometry.

**Results:**

The treatment resulted in a dose-related increase in the fraction of EPA and DHA in erythrocyte membranes and a dose-related decrease of other poly-unsaturated fatty acids, in particular omega-6 polyunsaturated fatty acids. Erythrocyte concentrations of saturated fatty acids remained unchanged throughout the experiment. Plasma concentrations of endocannabinoids and endocannabinoid-like mediators changed accordingly as those being downstream arachidonic acid decreased, downstream of the saturated palmitic and oleic acids remained unchanged while a downstream EPA metabolite increased.

**Conclusion:**

Increasing the omega-3 index by administering an omega-3 rich phospholipid extracted from krill oil did not alter the ratio of unsaturated vs. saturated fatty acids in the erythrocyte membranes but only the relative concentrations of unsaturated fatty acids, in particular unsaturated omega-6 fatty acids. Concentrations of saturated fatty acids remained unchanged.

## Background

The long-chain omega-3 fatty acids (FAs) eicosapentaenoic acid (EPA; C20:5n3) and docosahexaenoic acid (DHA; C22:6n3) are only to a very little extent produced by the human body but high levels can be obtained if they are supplied via the diet. EPA and DHA are found in marine sources, and a diet rich in fatty fish like mackerel, salmon and anchovies will secure a high intake of these FAs. A large number of scientific publications over the last few decades have demonstrated numerous beneficial health effects of a high intake of these long-chain omega-3 FAs (for a review, see [1]) and this has led to a significant industry delivering dietary supplements containing one or both of these two FAs. The source of the omega-3 s in these products is for the most part fatty fish, but various crustacean species like krill [2] and copepods [3], as well as algae [4], are also utilized.

The omega-3 index is often used to describe the levels of omega-3 FAs in the body. This index is defined as the percentage of EPA + DHA in erythrocyte membranes relative to the total amount of FAs in the membrane [5]. The analytical method commonly applied for the determination of the index in erythrocytes is gas chromatography following methylation of the fatty acid [6]. The index in humans is normally varying from about 2–3%, indicating a low EPA and DHA intake, up to about 11–12% which indicates a high intake [5].

Since an increased intake of long-chain omega-3 FAs will increase the fraction of EPA and DHA in the erythrocyte membrane it follows that the relative content of all or some of the other fatty acids and, subsequently, of their bioactive derivatives, must decrease, with a potential biological impact possibly beyond the mere alteration of the physiological function of those fatty acids. In the present study, increasing doses of a preparation of omega-3 rich phospholipids from krill oil was administered orally to non-human primates for a total of 12 weeks and the time course of the FA profiles in their erythrocytes was determined at baseline, during treatment and also after cessation of treatment. In addition to EPA and DHA, 22 FAs with an abundance varying from a fraction of a percent up to more than 20% at baseline were analyzed bi-weekly to investigate which of these were changed in the membranes when increasing amounts of EPA and DHA were administered. Furthermore, the concentrations of the two endocannabinoids, anandamide and 2-arachidonoylglycerol (2-AG), and four acylethanolamide endocannabinoid-like mediators, i.e. EPA-ethanolamide, DHA-ethanolamide, oleoylethanolamide and palmitoylethanolamide, were determined in plasma bi-weekly. The two endocannabinoids are downstream metabolites of arachidonic acid while the four endocannabinoid-like compounds are the ethanolamides of EPA, DHA, oleic acid and palmitic acid, respectively, [7] and it was expected that their plasma concentrations would reflect changes in the abundance of their parent FA in the membranes (see [8] and [9] for examples of such effects of krill preparations in humans and mice, respectively). Endocannabinoids and long-chain acetylethanolamides are known to have marked biological effects and changing the concentrations of these might potentially affect physiological processes in several organ systems since the receptors of these lipid mediators are widely distributed in the whole organism [10].

The data reported in this article originate from a study which also investigated the effects of the phospholipid preparation on several parameters known to be risk factors for development of cardiovascular disease; serum cholesterols, apolipoproteins, triglycerides, inflammatory biomarkers and diabetes biomarkers. Selected safety parameters were also analyzed. The results from this part of the study are reported separately [11].

## Results

**Fig. 1:**
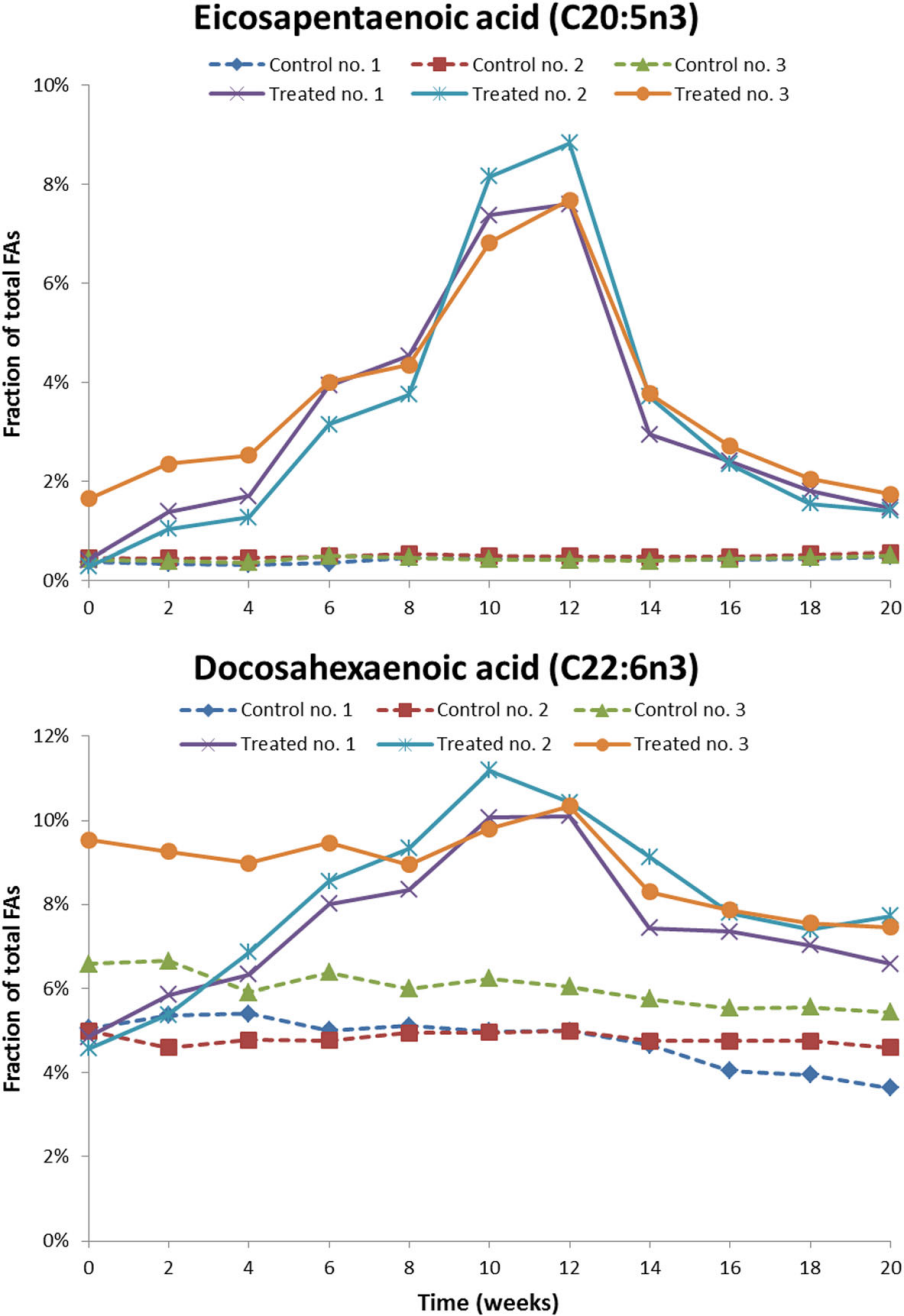
Individual concentrations of the main omega-3 fatty acids, EPA and DHA, in erythrocyte membranes, given as % of total fatty acids measured. *Dotted lines* represent control animals, *solid lines* represent treated animals which were given 50 mg/kg/day of the omega-3 rich phospholipids during week 1–4, 150 mg/kg/day during week 4–8 and 450 mg/kg/day during week 8–12. No treatment was given during week 12–20

**Fig. 2:**
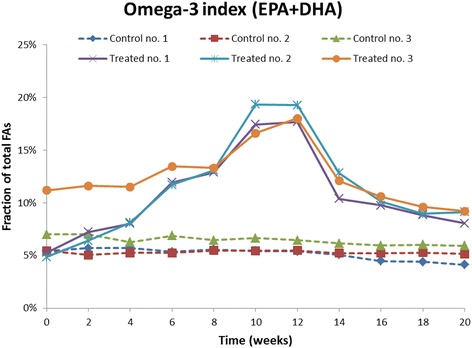
Individual values for Omega-3 index, defined as the sum of EPA and DHA as % of total fatty acids in the erythrocyte membrane. *Dotted lines* represent control animals, *solid lines* represent treated animals which were given 50 mg/kg/day of the omega-3 rich phospholipids during week 1–4, 150 mg/kg/day during week 4–8 and 450 mg/kg/day during week 8–12. No treatment was given during week 12–20

The relative fraction of the two main omega-3 fatty acids present in the phospholipid preparation administered to the non-human primates, EPA and DHA, increased dose-dependently in the treated animals (Fig. 1) while in the controls, only a minor change in the levels of these FAs was observed. For EPA, the levels in the treated animals increased from <1% at baseline to a mean of 8% at the end of treatment while DHA levels increased from a mean of 6% up to a mean of 10%. The omega-3 index (Fig. 2) increased from a mean of 7.2% at baseline to 9.2% at 4 weeks, i.e. at the end of the treatment period with the lowest dose of 50 mg/kg/day, to 13.1% at 8 weeks which was after end of treatment with the intermediate dose of 150 mg/kg/day and finally to 18.3% at the end of treatment with the high dose of 450 mg/kg/day at 12 weeks. During the 12-weeks of treatment with the vehicle control substance, which contained no EPA and DHA, the mean omega-3 index in the control group remained essentially unchanged as it only was reduced from 6.0% at baseline to 5.8% (Fig. 2).

Following cessation of treatment, the membrane concentration of EPA and DHA in the treated group declined consistent with first-order elimination kinetics (Fig. 1). The omega-3 index was reduced from a mean of 18.3% at the end of treatment to a mean of 11.8% 2 weeks into the wash-out period, and 8 weeks after end of treatment the mean omega-3 index in the treated group was 8.8% (Fig. 2). As seen from the figure the two fatty acids that constitute the omega-3 index, EPA and DHA, have different kinetics and the rate and extent of increase and decrease of the index are clearly affected differently by the two FAs.

The relative concentration of the omega-3 variant of docosapentaenoic acid (DPA; C22:5n3), which may be considered a metabolite of EPA, was higher than for EPA at baseline; the mean was 2.8% and increased to a mean of 4.5% at the end of the 12-week treatment (Fig. 5).

**Fig. 3:**
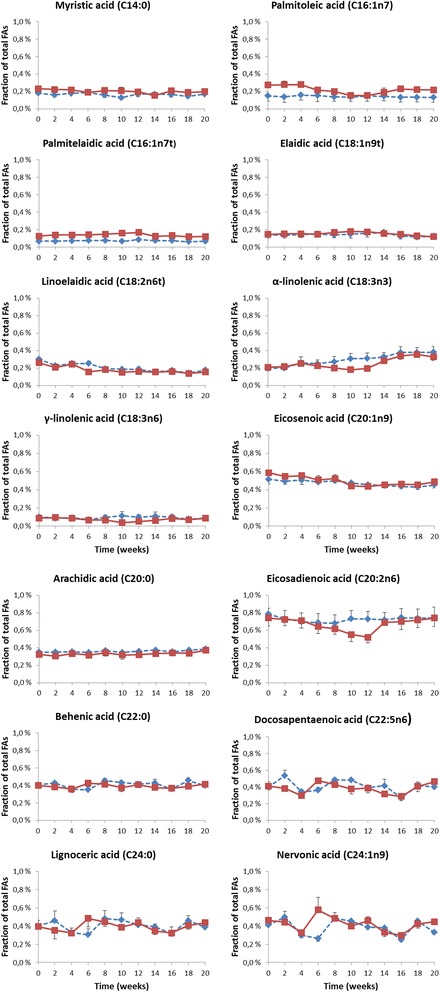
Mean concentrations and SEM of the less abundant (<1% at study start) fatty acids in erythrocyte membranes, given as % of total fatty acids measured, shown in the order of increasing chain length. *Blue dotted line* represents the control group, *red solid line* represents the treated group which was given 50 mg/kg/day of the omega-3 rich phospholipids during week 1–4, 150 mg/kg/day during week 4–8 and 450 mg/kg/day during week 8–12. No treatment was given during week 12–20

**Fig. 4:**
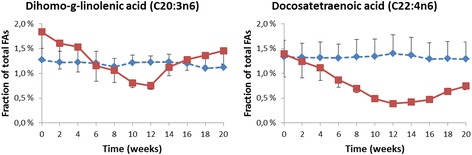
Mean concentrations and SEM of the median abundant (>1% but <2% at study start) fatty acids in erythrocyte membranes, given as % of total fatty acids measured, shown in the order of increasing chain length. *Blue dotted line* represents the control group, *red solid line* represents the treated group which was given 50 mg/kg/day of the omega-3 rich phospholipids during week 1–4, 150 mg/kg/day during week 4–8 and 450 mg/kg/day during week 8–12. No treatment was given during week 12–20

**Fig. 5:**
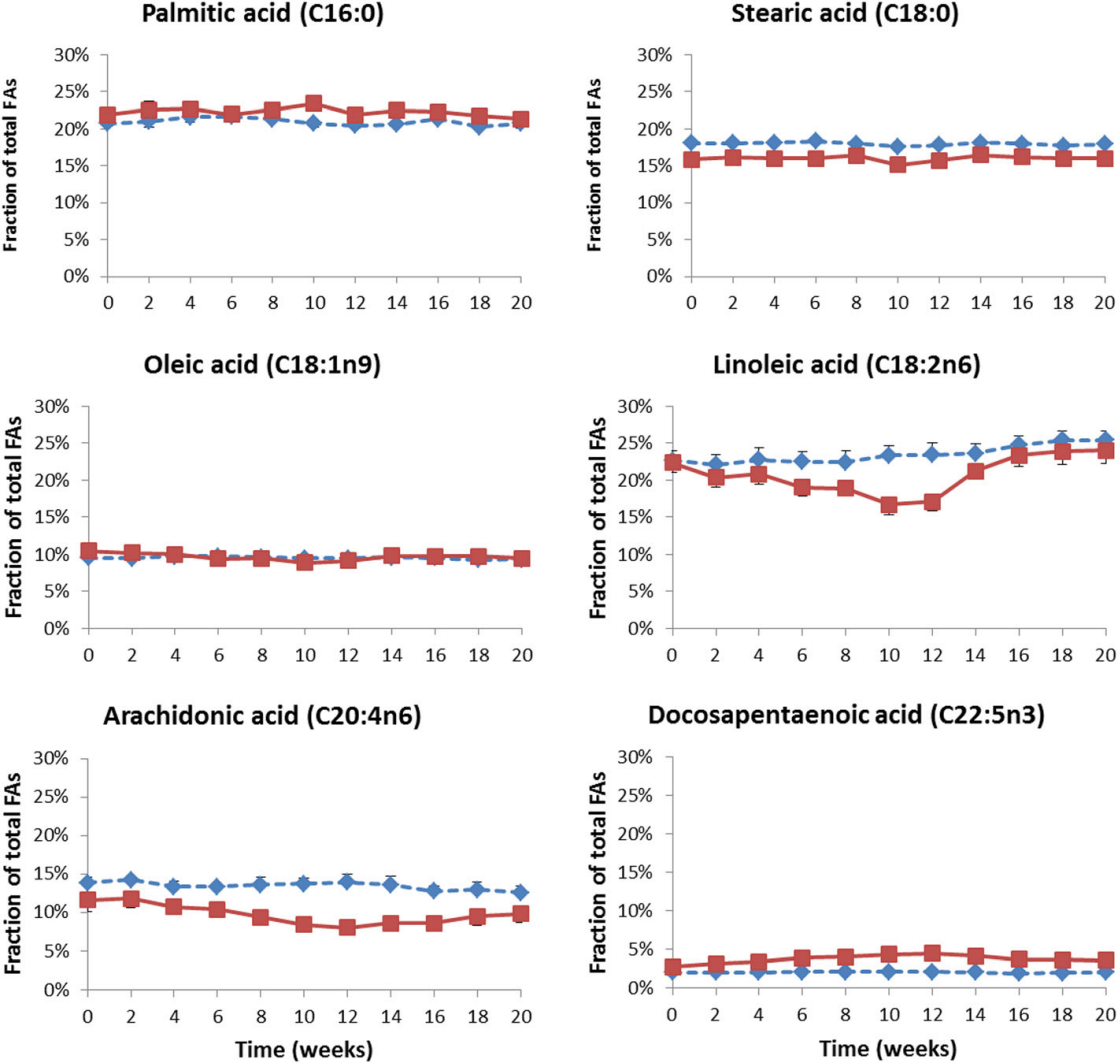
Mean concentrations and SEM of the most abundant (>2% at study start) fatty acids in erythrocyte membranes, given as % of total fatty acids measured, shown in the order of increasing chain length. *Blue dotted line* represents the control group, *red solid line* represents the treated group which was given 50 mg/kg/day of the omega-3 rich phospholipids during week 1–4, 150 mg/kg/day during week 4–8 and 450 mg/kg/day during week 8–12. No treatment was given during week 12–20

The mean fractions of 22 other fatty acids in erythrocyte membranes at each sampling time-point in the treated and control animals are presented in Table 1. These data are further visualized in Figs. 3, 4 and 5. The erythrocyte membrane fraction of the saturated FAs was apparently unchanged when the omega-3 FAs increased. The less abundant myristic acid (C14:0), arachidic acid (C20:0), behenic acid (C22:0) and lignoceric acid (C24:0) showed no consistent trend of increase or decrease (Fig. 3). All these FAs were present at <1% at baseline. The relative fraction of the major saturated FAs, palmitic acid (C16:0) and stearic acid (C18:0), which both were present in the membrane at a fraction of >15% at baseline, was unchanged throughout the 20 weeks the study lasted (Table 1 and Fig. 5). On the other hand, the relative fractions of most of the unsaturated omega-6 FAs were clearly altered during the study as the fraction of EPA and DHA increased, but with some exceptions.

**Table 1:** Mean fractions of fatty acids in erythrocyte membranes of the non-human primates (*n* = 3 in each of the two groups) at baseline, during and after treatment with the omega-3 rich phospholipid preparation, ranked according to increasing carbon chain-length and double-bonds

Fatty acid	Group	Baseline	Week after start of treatment									
			2	4	6	8	10	12	14	16	18	20
Myristic C14:0	Control	0.18%	0.16%	0.18%	0.19%	0.16%	0.13%	0.17%	0.17%	0.16%	0.14%	0.17%
	Treated	0.23%	0.22%	0.22%	0.19%	0.21%	0.21%	0.20%	0.15%	0.21%	0.19%	0.20%
Palmitic C16:0	Control	20.7%	21.0%	21.6%	21.7%	21.3%	20.8%	20.5%	20.6%	21.4%	20.3%	20.8%
	Treated	21.9%	22.5%	22.7%	22.0%	22.5%	23.4%	21.9%	22.5%	22.3%	21.8%	21.4%
Palmitoleic C16:1n7	Control	0.15%	0.14%	0.16%	0.15%	0.14%	0.14%	0.15%	0.14%	0.13%	0.13%	0.13%
	Treated	0.27%	0.28%	0.28%	0.22%	0.20%	0.15%	0.15%	0.19%	0.23%	0.22%	0.22%
Palmitelaidic C16:1n7t	Control	0.07%	0.07%	0.07%	0.07%	0.08%	0.06%	0.08%	0.07%	0.07%	0.06%	0.07%
	Treated	0.12%	0.14%	0.14%	0.14%	0.15%	0.16%	0.17%	0.12%	0.13%	0.12%	0.12%
Stearic C18:0	Control	18.1%	18.1%	18.1%	18.3%	18.0%	17.6%	17.8%	18.1%	18.0%	17.8%	17.9%
	Treated	15.9%	16.1%	16.0%	16.0%	16.4%	15.2%	15.7%	16.5%	16.2%	16.0%	16.0%
Oleic C18:1n9	Control	9.54%	9.49%	9.73%	9.72%	9.63%	9.50%	9.49%	9.59%	9.52%	9.30%	9.34%
	Treated	10.4%	10.2%	9.98%	9.43%	9.45%	8.88%	9.14%	9.80%	9.73%	9.72%	9.46%
Elaidic C18:1n9t	Control	0.14%	0.13%	0.14%	0.15%	0.14%	0.15%	0.16%	0.17%	0.12%	0.12%	0.12%
	Treated	0.14%	0.15%	0.15%	0.15%	0.17%	0.18%	0.17%	0.16%	0.14%	0.13%	0.12%
Linoleic C18:2n6	Control	22.7%	22.1%	22.7%	22.5%	22.4%	23.3%	23.4%	23.6%	24.8%	25.4%	25.4%
	Treated	22.4%	20.3%	20.8%	19.0%	18.9%	16.7%	17.1%	21.2%	23.4%	23.9%	24.1%
Linoelaidic C18:2n6t	Control	0.30%	0.23%	0.25%	0.25%	0.19%	0.19%	0.18%	0.16%	0.17%	0.14%	0.17%
	Treated	0.26%	0.21%	0.25%	0.16%	0.18%	0.15%	0.16%	0.15%	0.16%	0.14%	0.15%
alpha-linolenic C18:3n3	Control	0.20%	0.20%	0.26%	0.25%	0.27%	0.31%	0.31%	0.33%	0.38%	0.38%	0.38%
	Treated	0.21%	0.22%	0.25%	0.22%	0.20%	0.18%	0.20%	0.28%	0.34%	0.35%	0.33%
gamma-linolenic C18:3n6	Control	0.10%	0.09%	0.09%	0.07%	0.10%	0.11%	0.10%	0.11%	0.10%	0.08%	0.09%
	Treated	0.09%	0.09%	0.09%	0.07%	0.07%	0.04%	0.05%	0.06%	0.08%	0.07%	0.09%
Arachidic C20:0	Control	0.35%	0.35%	0.35%	0.35%	0.37%	0.35%	0.36%	0.37%	0.35%	0.37%	0.38%
	Treated	0.32%	0.31%	0.33%	0.32%	0.34%	0.32%	0.32%	0.33%	0.34%	0.34%	0.37%
Eicosenoic C20:1n9	Control	0.52%	0.49%	0.51%	0.49%	0.50%	0.47%	0.45%	0.45%	0.44%	0.43%	0.45%
	Treated	0.59%	0.55%	0.55%	0.51%	0.52%	0.44%	0.44%	0.46%	0.46%	0.45%	0.49%
Eicosadienoic C20:2n6	Control	0.79%	0.73%	0.70%	0.69%	0.68%	0.73%	0.73%	0.72%	0.75%	0.74%	0.74%
	Treated	0.74%	0.72%	0.71%	0.64%	0.62%	0.55%	0.52%	0.69%	0.70%	0.72%	0.74%
Dihomo-g-linolenic C20:3n6	Control	1.27%	1.22%	1.23%	1.21%	1.13%	1.22%	1.23%	1.23%	1.20%	1.10%	1.12%
	Treated	1.84%	1.61%	1.53%	1.15%	1.06%	0.81%	0.75%	1.12%	1.28%	1.36%	1.46%
Arachidonic C20:4n6	Control	13.9%	14.3%	13.4%	13.4%	13.7%	13.8%	13.9%	13.6%	12.8%	13.0%	12.6%
	Treated	11.7%	11.9%	10.8%	10.5%	9.41%	8.49%	8.10%	8.63%	8.63%	9.53%	9.88%
Behenic C22:0	Control	0.40%	0.43%	0.36%	0.35%	0.45%	0.43%	0.41%	0.43%	0.36%	0.46%	0.40%
	Treated	0.40%	0.38%	0.36%	0.42%	0.41%	0.37%	0.41%	0.38%	0.37%	0.39%	0.41%
Docosatetraenoic C22:4n6	Control	1.33%	1.33%	1.32%	1.31%	1.33%	1.34%	1.40%	1.36%	1.29%	1.30%	1.29%
	Treated	1.39%	1.24%	1.11%	0.87%	0.70%	0.49%	0.39%	0.42%	0.47%	0.64%	0.74%
Docosapentaenoic C22:5n3	Control	2.10%	2.07%	2.09%	2.13%	2.13%	2.14%	2.12%	2.04%	1.95%	2.02%	2.06%
	Treated	2.75%	3.18%	3.42%	3.93%	4.07%	4.36%	4.53%	4.16%	3.74%	3.65%	3.63%
Docosapentaenoic C22:5n6	Control	0.40%	0.54%	0.34%	0.36%	0.49%	0.48%	0.39%	0.41%	0.27%	0.42%	0.40%
	Treated	0.41%	0.38%	0.30%	0.48%	0.43%	0.38%	0.39%	0.31%	0.29%	0.41%	0.46%
Lignoceric C24:0	Control	0.40%	0.46%	0.33%	0.30%	0.48%	0.47%	0.41%	0.39%	0.31%	0.46%	0.39%
	Treated	0.39%	0.35%	0.32%	0.48%	0.44%	0.39%	0.44%	0.35%	0.32%	0.41%	0.44%
Nervonic C24:1n9	Control	0.41%	0.50%	0.30%	0.26%	0.48%	0.45%	0.39%	0.38%	0.25%	0.45%	0.33%
	Treated	0.46%	0.44%	0.33%	0.58%	0.48%	0.40%	0.46%	0.33%	0.30%	0.42%	0.45%
Dose (mg phospholipids/kg bw/day)			50		150		450		0			
Dose (mg [EPA/DHA]/kg bw/day)			9.35/5.48		28.0/16.4		84.1/49.3		0/0			

Doses of administered phospholipids and of EPA and DHA are shown at the end of the table

A considerable reduction was seen for arachidonic acid (C20:4n6) which was reduced from a mean of 11.7 to 8.1% at 12 weeks, i.e. a reduction of approximately 30% from baseline (Fig. 5). During the 8-week wash-out period a clear rebound effect was observed and at the end of this period, the arachidonic acid level was only 6% lower than at baseline. Another abundant unsaturated FA, linoleic acid (C18:2n6) with a baseline level of 22%, was reduced down to 17.1% after the 12 week treatment (Fig. 5), a relative reduction of 27%. Oleic acid (C18:1n9) had a mean baseline level of 10.4% in the treated group and was reduced to a mean of 9.1% at the end of treatment (Fig. 5), which represents a relative reduction of about 12%. The fraction of both these FAs approached their baseline levels during the 8 week wash-out period. Alpha-linolenic acid (C18:3n3) and gamma-linolenic acid (C18:3n6), both polyunsaturated FAs present at very low levels in the membranes, were both reduced during treatment with the long-chain omega-3 rich phospholipids (Fig. 3). For all FAs being reduced during treatment, the extent of reductions were apparently depending on the dosages of EPA and DHA and the reductions were always most prominent during weeks 8–12 of treatment where the highest dose of 450 mg phospholipids/kg/day was administered.

The two medium-abundant unsaturated omega-6 FAs, dihomo-g-linolenic acid (C20:3n6) and docosatetraenoic acid (C22:4n6), had the largest relative reduction of all FAs measured. The former FA was reduced by almost 60%; from a mean fraction of 1.84% at baseline to 0.75% at 12 weeks, while the latter was reduced by 72%, i.e. from a mean fraction of 1.39% at baseline to a mean of 0.39% at 12 weeks (Fig. 4).

Three trans-FAs were measured in the erythrocyte membranes, palmitelaidic acid (C16:1n7t), elaidic acid (C18:1n9t) and linoelaidic acid (C18:2n6t). None of these were abundant in the membranes as all three had a relative fraction of around 0.2% at baseline, but they appeared unchanged throughout the 20 weeks the study lasted (Fig. 3).

Generally, for all FAs which changed their relative concentration in the erythrocyte membranes, a clear rebound effect was observed and most of them were close to the baseline levels 8 weeks after cessation of treatment with the omega-3 rich phospholipid preparation.

The results from the analyses of endocannabinoids and endocannabinoid-like compounds in plasma are summarized in Table 2. As indicated by the standard error of the means given in the table, the plasma concentrations of these compounds showed a marked variation between the animals and for some of the animals, including the controls, the concentrations also varied considerably from one time-point to the next. However, when adjusting the mean of the three animals in the treated group with the mean for the control group, the pattern for each of the endocannabinoid-type compounds in the treated group was generally consistent with the changes in the erythrocyte concentrations of the related FAs. Anandamide (arachidonoylethanolamide) and 2-AG are downstream derivatives of arachidonic acid and showed a decreasing trend in parallel with the decreased fraction of their parent FA in erythrocytes. EPA-ethanolamide, an endocannabinoid-like mediator ultimately formed from EPA, increased in parallel to the increased fraction of EPA in erythrocytes. DHA-ethanolamide, ultimately formed from DHA, showed, however, no consistent trend of increase or decrease despite the observed increase in the fraction of DHA in the erythrocyte membranes. Two other endocannabinoid-like compounds, palmitoylethanolamide and oleoylethanolamide, ultimately formed from the saturated FAs palmitic acid and oleic acid, respectively, did not show any trend of change in the plasma concentration which is in line with the unchanged erythrocyte fraction of their parent FAs. Similar to the FAs in the erythrocyte membranes, the plasma concentration of the affected FA metabolites returned towards the baseline or even beyond the baseline levels after cessation of treatment with the phospholipid preparation.

**Table 2:** Concentrations (pmol/ml) of the endocannabinoid-type compounds measured in plasma from the non-human primates (*n* = 3 in each of the two groups) at baseline, during and after treatment with increasing doses of the omega-3 rich phospholipid preparation

Endocannabinoid-type compound	Group	Value	Baseline	Week after start of treatment									
				2	4	6	8	10	12	14	16	18	20
Anandamide	Control	Mean	4.24	4.80	5.60	4.31	4.82	4.66	6.02	6.19	2.82	5.19	5.15
		SEM	2.00	1.18	2.17	0.93	0.80	0.91	3.84	2.65	0.77	1.38	1.67
	Treated	Mean	3.07	3.40	2.86	2.98	2.86	3.34	2.28	3.64	4.25	4.05	4.44
		SEM	0.52	1.54	0.93	0.56	0.92	1.35	0.97	1.35	1.69	1.53	0.43
2-arachidonoylglycerol	Control	Mean	14.3	21.0	23.1	21.9	27.8	20.7	28.6	22.7	19.0	18.6	22.4
		SEM	5.5	13.8	15.9	11.1	19.2	15.2	20.6	12.3	11.8	11.3	15.5
	Treated	Mean	16.8	19.2	13.3	9.14	11.7	4.42	8.48	15.0	26.5	16.5	26.4
		SEM	6.4	9.2	7.9	1.12	3.3	1.60	4.20	10.0	19.1	9.1	11.3
EPA ethanolamide	Control	Mean	1.36	1.45	0.91	1.25	0.22	0.57	1.00	1.07	2.07	2.04	2.84
		SEM	0.49	0.29	0.16	0.31	0.07	0.20	0.07	0.71	0.84	0.62	1.06
	Treated	Mean	0.47	1.04	1.05	1.13	0.85	1.00	1.07	0.58	1.34	1.55	0.63
		SEM	0.13	0.48	0.34	0.26	0.24	0.17	0.26	0.22	0.93	0.51	0.35
DHA ethanolamide	Control	Mean	1.23	4.67	5.17	6.64	4.95	4.63	4.61	2.28	3.89	5.82	4.80
		SEM	0.29	1.35	2.09	1.95	2.45	2.03	2.06	1.10	1.45	2.49	1.91
	Treated	Mean	3.06	6.63	8.03	7.24	6.60	7.41	4.96	3.49	5.57	5.97	6.76
		SEM	1.68	3.17	2.34	1.52	1.59	1.12	0.89	0.39	1.30	2.83	2.93
Palmitoyl ethanolamide	Control	Mean	54.7	64.7	58.3	94.7	53.5	71.9	89.9	30.8	24.7	59.3	120
		SEM	4.70	10.3	15.3	30.5	12.7	11.2	7.80	4.70	1.50	20.8	36.3
	Treated	Mean	66.2	62.5	65.6	86.1	58.6	48.4	69.1	78.8	39.5	92.0	120
		SEM	19.2	11.9	14.7	17.3	13.9	9.20	2.40	23.7	8.3	31.5	47.9
Oleoyl ethanolamide	Control	Mean	24.2	24.3	19.0	20.3	30.5	28.5	14.0	29.1	17.6	29.3	31.0
		SEM	4.44	1.50	2.25	2.23	1.55	2.65	1.35	5.26	4.10	4.60	2.28
	Treated	Mean	24.2	22.3	16.0	20.3	24.8	29.9	14.4	36.9	18.0	39.7	45.5
		SEM	3.63	6.86	2.05	2.49	6.63	7.76	2.54	15.01	3.03	14.54	9.89
Dose (mg phospholipids/kg bw/day)				50		150		450		0			
Dose (mg [EPA/DHA]/kg bw/day)				9.35/5.48		28.0/16.4		84.1/49.3		0/0			

Doses of phospholipids and of EPA and DHA are shown at the end of the table

## Discussion

The main finding of the present investigation is that when the fractions of the long-chain omega-3 FAs EPA and DHA in erythrocyte membranes increased as a result of oral treatment with an omega-3 rich phospholipid preparation extracted from krill oil, the FAs reduced were only other unsaturated FAs and in particular omega-6 FAs. None of the saturated FAs changed their relative presence in the membranes, whether they were abundant or not. The relative fraction of monounsaturated FAs seems to be less affected than polyunsaturated ones. Also the measured trans FAs, although present only in very low fractions, appeared unchanged during the treatment period. It is of note that the relative fractions of EPA and DHA increased markedly as a result of treatment, and the omega-3 index, representing the sum of EPA and DHA in the membranes, increased from 7.3% at baseline to 18.2% at the end of the 12-week treatment. The omega-3 dose-levels applied in this study are relevant in a human setting since the dose-levels of EPA + DHA used, when converted to human equivalent dose (HED) levels, amount to 300, 860 and 2580 mg/day to an adult human of 60 kg. The HED is calculated based on body surface area and obtained by dividing the dose in the monkeys, given as mg/kg, by 3.1 and multiplying by the human body weight [12].

Our data are in line with the results from the study by Katan et al. [13], who gave three doses of fish oil to adults and measured omega-3 fatty acids as well as two omega-6 fatty acids, linoleic and arachidonic acids, in erythrocytes. The omega-3 fatty acids increased dose-dependently while the two omega-6 fatty acids were reduced dose-dependently. These authors did not report concentrations of any saturated fatty acids. However Ramprasath and co-workers compared the increase in omega-3 index, omega-6:omega-3 ratio, total saturated fatty acids, total monounsaturated fatty acids and total omega-6 fatty acids after krill oil and fish oil after 4 weeks of treatment and found that total saturated fatty acids were unchanged despite a clear increase in omega-3 index and a reduction in the omega-6:omega-3 ratio. These authors did not specify the levels of individual fatty acids except for EPA, DHA and DPA [14].

The potential consequences of these changes in the composition of the cell membranes induced by the administration of EPA and DHA were not investigated in this study but the assumption is that the balance between saturated and unsaturated FAs has implications for membrane fluidity and thereby cellular function [15]. Therefore, the observation that the membrane content of saturated vs. unsaturated FAs is relatively unaffected by the introduction of EPA and DHA is interesting. Of note are also the changes in the plasma concentrations of endocannabinoids and endocannabinoid-like mediators, which generally followed the changes in erythrocyte FA abundance. This was not surprising since these mediators are ultimately derived from membrane phospholipids containing the corresponding fatty acids in either the *sn*-1 (in the case of acylethanolamides) or *sn*-2 (in the case of 2-acylglycerols such as 2-AG) positions. Due to the various physiological effects of the endocannabinoids, in particular their regulatory effects on appetite, food intake, adipose tissue and ectopic fat accumulation and insulin sensitivity [16], a reduction of the relative abundance of their precursors and, subsequently, of their biosynthesized amounts, may have significant biological implications. Thus, the trend for the reduction in arachidonoylethanolamide (anandamide) and 2-AG levels may explain in part some of the beneficial metabolic effects observed with the krill oil preparation [11]. However, also EPA and DHA-derived ethanolamides are emerging as bona fide non-endocannabinoid mediators with potential anti-inflammatory actions [17] and we found here that EPA-ethanolamide, which is ultimately derived from the long chain omega-3 FA whose levels increased the most and most consistently in our study, i.e. EPA, was also increased following treatment with krill oil phospholipids.

The finding that the main effect of a high intake of omega-3 FAs is a progressive change in the relative abundance of the polyunsaturated FAs and not a displacement of saturated FAs from the cell membrane is also supporting the theory that the reported anti-inflammatory effects of omega-3 FAs may, at least partly, be due to a reduced membrane concentration of arachidonic acid which in turn should lead to reduced production of proinflammatory arachidonic acid metabolites like prostaglandins, leukotriens and thromboxanes [18]. Our data show that the fraction of arachidonic acid in the membranes was reduced by about 30% as a result of the 12-week treatment with increasing doses of the omega-3 rich phospholipids. An additional aspect is the effects of the so-called ‘specialized proresolving mediators’; the resolvins, protectins and maresins, all molecules biosynthesized from long-chain omega-3 FAs and shown to stimulate proresolving cellular processes such as inhibition of neutrophil infiltration, enhancement of macrophage phagocytosis of bacteria and efferocytosis of cellular debris, and reduction of inflammatory pain through specific G-protein coupled receptors [19]. The proresolving effects of these specialized proresolving mediators would be expected to work in concert with the decreased proinflammatory effects caused by a reduced production of arachidonic acid metabolites and together, these two mechanisms might to a large extent explain the beneficial effects the long-chain omega-3 FAs have on inflammation.

The long-chain omega-3 FAs used in the present study were administered to the animals as phospholipids. The most common form of omega-3 FAs in the human diet is, however, triglycerides since omega-3 FAs in fish are mostly in this chemical form [2]. Our study did not compare the outcome of administration of omega-3 FAs as phospholipids and as triglycerides and the results obtained in our study should therefore not be generalized as effects of any omega-3 preparation used. Since omega-3 FAs in cell membranes mostly are incorporated in phospholipids, it might be that the chemical form administered has implications on how the FAs are distributed in cell membranes. There has been some debate in the literature whether delivery of long-chain omega-3 FAs as phospholipids, which is the case when the source is krill oil, is more efficient for building up the omega-3 index than when delivering these FAs as triglycerides like in most fish oils. A recent article by Yurko-Mauro and co-workers concluded that giving EPA and DHA in either ethyl ester, triglyceride or phospholipid form for 4 weeks created similar plasma and erythrocyte levels of the FAs [20]. However, other literature has indicated a more efficient uptake of these omega-3 FAs when given as krill oil than with fish oil [21, 22], resulting in higher omega-3 indices after dose-corrections. From a general point of view one would expect FAs to be absorbed almost completely from forms that are natural, i.e. triglycerides and phospholipids, since the digestion of fat in the gastrointestinal tract is tailor-made for optimal absorbtion of this type of nutrients [23]. It is of note, however, that Ramprasath and coworkers have published their observation that the content of phospholipids in krill oil may have an impact on the bioavailability of the omega-3 FAs [24]. Given that the preparation tested in our study was highly purified with a phospholipid content of >98%, this may have led to a higher increase in the omega-3 index than would a normal krill-oil preparation with an approximately 40% phospholipid content, provided comparable doses of omega-3 FAs.

The omega-3 index determined in erythrocyte membranes is well correlated with omega-3 levels in other tissues, in particular heart tissue [25]. A high omega-3 index, preferably above 8%, has been shown to be strongly correlated with a reduced risk of sudden cardiac death [26]. Furthermore, a low omega-3 index has also been shown to correlate with an increased risk for disease of the central nervous system like depression [27] and an increased intake of long-chain omega-3 FAs has been suggested as a treatment strategy for maintaining cognitive health into older age [28].

An obvious limitation of our study is the relatively low number of animals included in each of the control and treated groups, which precludes a statistical analysis of the data. However, given that the species used is a non-human primate, animal ethics suggests that the number used should be as low as possible, provided meaningful data can be obtained. Animals fed a standard diet over long periods of time, like those in the present study, will generally have much less variation in their baseline FA profiles than humans who have a variable diet and therefore more heterogeneous FA profiles. This was confirmed by the low variation in erythrocyte FA abundance between the animals in the control group. Also the consistent changes in the FAs in the treated animals indicate that the trends observed are reliable.

## Conclusions

Feeding non-human primates with increasing doses of an omega-3 rich phospholipid preparation extracted from krill oil caused a dose-related increase in the fraction of the long-chain omega-3 FAs EPA and DHA in erythrocyte membranes and a dose-related decrease of other polyunsaturated FAs, and in particular omega-6 polyunsaturated FAs. Erythrocyte concentrations of saturated fatty acids remained unchanged throughout the experimental period. Hence, increasing the omega-3 index does apparently not alter the ratio of unsaturated vs. saturated FAs but only the relative concentrations of unsaturated FAs. The plasma concentrations of six endocannabinoid-type mediators changed accordingly, as those being downstream arachidonic acid decreased, those downstream the saturated palmitic and oleic acids remained unchanged, and a downstream EPA metabolite increased.

## Methods

### Test and control items

The product tested in the current investigation is a preparation of purified (>98% pure) phospholipids extracted from krill-oil, rich in the long-chain omega-3 fatty acids eicosapentaenoic acid (EPA; C20:5n3) and docosahexaenoic acid (DHA; C22:6n3). The final product was formulated to improve its floating properties, thus making it easier to administer, and had the following composition: phospholipids 84% (840 mg/ml), polyethylene glycol with MW 400 (PEG400) 12.5% and ethanol 3.5%. The abundance of FAs in the end-product was analyzed by methylation of the FAs followed by gas-chromatography and 25 fatty acids were detected and quantified. The EPA and DHA contents were 15.7% (157 mg/g) and 9.2% (92 mg/g), respectively. In addition, five other FAs were present in the end-product with an abundance of more than 1% (10 mg/g): myristic acid (C14:0) 1.1%, palmitic acid (C16:0) 13.2%, oleic acid (C18:1n9) 2.1%, cis-vaccenic acid (C18:1n11) 3.1% and stearidonic acid (C18:4n3) 1.3%. All other detected FAs in the end-product were below 1%. The formulation used has been proven stable for at least 6 months.

The control substance was designed to be a vehicle control and was made similarly as the test substance but by substituting the phospholipids with water. Therefore, the control substance contained 84% water, 12.5% PEG400 and 3.5% ethanol.

### Animals

Six type-2 diabetic and dyslipidemic cynomolgus monkeys (*Macaca fascicularis*) were selected for the study, after screening a total of 34 animals. Inclusion criteria were predefined and were related to diabetes parameters (HbA1c, glucose and insulin) and blood lipid levels (triglycerides, total cholesterol, low-density lipoprotein cholesterol (LDL-c) and high-density lipoprotein cholesterol (HDL-c)). The results from the screening as well as details of housing conditions and care are described in [11]. Vital data of the monkeys included in the study are given in Table 3.

**Table 3:** Vital data of the six non-human primates included in the study

Group	Animal ID	Sex	Age (years)	Bodyweight (kg)
Control				
	Control no. 1	M	14	9.22
	Control no. 2	M	15	10.0
	Control no. 3	F	13	4.32
Treated				
	Treated no. 1	M	14	8.30
	Treated no. 2	F	21	7.34
	Treated no. 3	M	20	10.8

The animals were fed a standard monkey diet containing crude protein (≥16%), crude fat (≥4%), moisture (≤10%), ash (≤7%), fiber (≤4%), calcium (0.8–1.2%) and phosphorus (0.6–0.8%). The content of omega-3 fatty acids in the diet had not been determined by the supplier (Beijing Keao Xieli Feed Co. Ltd., Beijing, P. R. China). Care and use of the animals were conducted in accordance with all applicable assessment and accreditation of the laboratory animal care (AAALAC) regulations and guidelines. Crown bioscience institutional animal care and use committee (IACUC) approved all animal procedures used in the study. All the procedures related to handling, care and treatment of the animals in this study were performed according to the guidelines approved by AAALAC. After completion of the study the animals were returned to the stock of diabetic monkeys held at Crown Biosciences for a recovery period and following this they were allowed to be used in other studies.

### Dosing

The test and control items were dosed once per day. Just prior to administration the product was warmed up to 35 °C and mixed with lukewarm water in a syringe in the ratio 1:5 to become an emulsion. Immediately thereafter, the emulsion was administered by connecting a naso-/oral gastric tube to the syringe and inserting it into the stomach. To ascertain that the entire dose was given the tube was flushed with lukewarm water. The control item was handled in the same way as the test item.

The control and test items were given by gavage, 1 h after the first feeding of the day. The control article was administered at the same volume/kg as the test article. For the first 4 weeks, a phospholipid dose of 50 mg/kg/day was given, the next 4 weeks the dose was 150 mg/kg/day and for the last 4 weeks 450 mg/kg/day. These phospholipid doses resulted in EPA/DHA doses of 9.35/5.48 mg/kg/day, 28.0/16.4 mg/kg/day and 84.1/49.3 mg/kg/day, respectively. Dosing volumes were based on body weights recorded bi-weekly. When converted to human equivalent dose (HED) these doses were equivalent to approximately 1, 3 and 10 g phospholipids/day, respectively, to an adult person of 70 kg.

### Blood sampling

Blood was sampled bi-weekly and centrifuged at 3000–3500 rpm for 10–15 min at room temperature. After discarding the plasma and buffy coat a minimum of 0.25 ml red blood cells (RBC) from the middle of the RBC layer was collected and transferred to a well-labeled storage cryovial. The samples were frozen at −80 °C until being shipped out on dry ice for analysis.

### Analysis

The analysis of fatty acids in the erythrocyte membranes was performed by OmegaQuant, Sioux Falls, SD USA, by gas chromatography (GC) with flame ionization detection [6]. OmegaQuant is a Clinical Laboratory Improvement Amendments (CLIA)-certified laboratory and the assay for fatty acid analysis has been validated and standardized [29]. The company has not published all details of the method and the validation of it since they consider this information proprietary. However, the coefficient of variation (CV) of the method for EPA, DHA and arachidonic acid is reported to be between 5 and 8% for all three fatty acids [29] and for the omega-3 index the CV is reported to be <5% [6].

Unwashed, packed erythrocytes were transferred to a screw-cap glass vial. Methanol containing 14% boron trifluoride (Sigma-Aldrich, St. Louis, MO) and hexane (EMD Chemicals, USA) were added sequentially. The vial was briefly vortexed and heated in a hot bath at 100 °C for 10 min. After cooling, HPLC grade water was added, the tubes were recapped, vortexed and centrifuged to separate layers. An aliquot of the hexane layer was transferred to a GC vial. GC was carried out using a GC2010 Gas Chromatograph (Shimadzu Corporation, Columbia, MD) equipped with an SP2560, 100-m fused silica capillary column (0.25 mm internal diameter, 0.2 um film thickness; Supelco, Bellefonte, PA).

FAs were identified by comparison with a standard mixture of fatty acids characteristic of RBC (GLC OQ-A, NuCheck Prep, Elysian, MN) which was also used to determine individual fatty acid calibration curves. In addition to EPA (C20:5n3) and DHA (C22:6n3), the following 22 fatty acids were quantified: myristic (14:0), palmitic (16:0), palmitoleic (C16:1n7), palmitelaidic (C16:1n7t), stearic (18:0), oleic (C18:1n9), elaidic (C18:1n9t), linoleic (C18:2n6), linoelaidic (C18:2n6t), alpha-linolenic (C18:3n3), gamma-linolenic (C18:3n6), arachidic (20:0), eicosenoic (C20:1n9), eicosadienoic (C20:2n6), dihomo-g-linolenic (C20:3n6), arachidonic (C20:4n6), behenic (22:0), docosatetraenoic (C22:4n6), docosapentaenoic (C22:5n3 and C22:5n6), lignoceric (24:0) and nervonic (C24:1n9) acids. The chromatographic conditions used in this study were sufficient to isolate the C16:1trans isomers and the C18:2, 9 t-12c, 9 t-12 t, and 9c-12 t isomers, which are reported as C18:2n6t. However, each individual C18:1 trans molecular species (i.e., C18:16 thru 13) could not be segregated but appeared as two blended peaks that eluted just before oleic acid. The areas of these two peaks were summed and referred to a C18:1 trans.

FA composition was expressed as a percent of total identified fatty acids. The omega-3 index was calculated as the sum of percent fraction of EPA (C20:5n3) and DHA (C22:6n3).

The concentrations of the endocannabinoids anandamide (AEA), 2-arachidonoylglyceride (2-AG), EPA ethanolamide (EPAEA), DHA ethanolamide (DHAEA), palmitoyl ethanolamide (PEA) and oleoyl ethanolamide (OEA) in plasma were analyzed as described previously [30]. In brief, samples were dounce-homogenized and extracted with chloroform/methanol/Tris-HCl 50 mM pH 7.5 (2:1:1, v/v) containing internal deuterated standards for AEA, 2-AG, PEA and OEA quantification by isotope dilution (10 pmol of [^2^H]_8_AEA and 50 pmol of [^2^H]_5_2AG, [^2^H]_4_ PEA, [^2^H]_4_ OEA (Cayman Chemicals, MI, USA). Quantification of EPAEA and DHAEA was done by using deuterated AEA as internal standard. The lipid-containing organic phase was dried down, weighed and pre-purified by open bed chromatography on silica gel. Fractions were obtained by eluting the column with 99:1, 90:10 and 50:50 (v/v) chloroform/methanol. The 90:10 fraction was used for AEA, 2-AG, PEA and OEA quantification by liquid chromatography-atmospheric pressure chemical ionization-mass spectrometry (LC-APCI-MS), as previously described and using selected ion monitoring at M + H^+^ values for the four compounds and their deuterated homologues, as described in [31, 32]. Pilot experiments showed that the ratios between the peak areas of the M + H^+^ ions for each of the non-deuterated compounds and those of a known amount (10 or 50 pmol) of the corresponding deuterated compounds (or of deuterated AEA in the case of EPAEA and DHAEA) varied linearly with the concentration of the non-deuterated compounds, over the 0.1–1000 pmol range, and therefore were suitable for quantification. The CV of these methods have also been described in previous papers (see [32] and [33] for recent reviews).

### Data presentation and statistics

For the omega-3 FAs EPA and DHA, and for the omega-3 index, individual values for each of the 6 animals are presented graphically. For the other 22 FAs, mean values for the control and treated groups are tabulated and mean and SEM values presented graphically. For the endocannabinoids, mean and SEM values for the control and treated groups are tabulated. Mean rather than median values were used to describe the central tendency for each group, despite the small group size, since this also made it possible to give SEM as a measure of spread. Due to the low number of animals in each of the control and treated groups, no comparison between groups based on statistical criteria was performed.

## Abbreviations

2-AG: 2-Arachidonoylglycerol
ANA: Anandamide
bw: Body weight
DHA: Docosahexaenoic acid
DHAEA: DHA ethanolamide
EPA: Eicosapentaenoic acid
EPAEA: EPA ethanolamide
FA: Fatty acid
OEA: Oleoyl ethanolamide
PEA: Palmitoyl ethanolamide
RBC: Red blood cells
